# The association between maternal exposure to secondhand smoke during pregnancy and their children’s cerebral palsy, Shandong, China

**DOI:** 10.18332/tid/127872

**Published:** 2020-10-21

**Authors:** Songtao Ren, Shaohua Xie, Xuri Li, Guofeng Li, Yan Wang, Weidong Liu, Li Wang

**Affiliations:** 1 Department of Neurosurgery, Liaocheng People’s Hospital, Liaocheng, People’s Republic of China; 2 School of Medicine, Shandong University, Jinan, People’s Republic of China; 3 Medical College, Liaocheng University, Liaocheng, People’s Republic of China; 4 Department of Pediatrics, Liaocheng People’s Hospital, Liaocheng, People’s Republic of China; 5 Department of Gynecology and Obstetrics, Qingdao Hiser Medical Group, Qingdao, People’s Republic of China; 6 Department of Physical Medicine, Liaocheng Nο. 4 People’s Hospital, Liaocheng, People’s Republic of China; 7 Department of Medical Record, Liaocheng No. 4 People’s Hospital, Liaocheng, People’s Republic of China; 8 Department of Gynecology, Liaocheng People’s Hospital, Liaocheng, People’s Republic of China; 9 Metabolism Group, Research Unit of Molecular Epidemiology, Institute of Epidemiology, Helmholtz Zentrum München, German Research Center for Environmental Health, Munich, Germany

**Keywords:** pregnancy, secondhand smoke, cerebral palsyReceived, 10 March 2020Revised, 13 August 2020

## Abstract

**INTRODUCTION:**

Tobacco use poses a threat to the health of pregnant women and their children. Our study assessed the association between maternal exposure to secondhand smoke (SHS) during pregnancy and children’s cerebral palsy (CP) in Shandong, China.

**METHODS:**

In our observational study, 5067 mother–child pairs were included from Shandong Province, China. Mothers filled in questionnaires about exposure to SHS during pregnancy. Statistical analysis and logistic regression models were built in R program to estimate the association in adjusted odds ratio (AOR) between SHS exposure during pregnancy and risk of children’s CP, after adjustment for potential confounders including delivery mode and baby’s birthweight.

**RESULTS:**

Exposure to SHS was noted among 3663 (72.3%) of the 5067 non-smoking mothers during their pregnancy. Of the 239 CP children within the study, 192 (80.3%) were exposed to SHS during pregnancy. Children born to mothers exposed to SHS during pregnancy had a higher risk of CP (AOR=1.44; 95% CI: 1.02–2.04) than those born to non-exposed mothers, the risk increased by exposure time in the logistic regression model. The association between SHS exposure during pregnancy and CP children remained significant when adjusting for delivery mode and infant’s birthweight due to their significant association with CP, with an AOR of 1.46 (95% CI: 1.13–1.91) for 1–4 days/week and 1.63 (95% CI: 1.22–2.01) for 5–7 days/week exposure to SHS.

**CONCLUSIONS:**

Our study suggests that maternal exposure to secondhand smoke during pregnancy is associated with children’s CP. Future preventive interventions of CP should include strategies that target the antenatal women who are exposed to SHS.

## INTRODUCTION

The use of tobacco is considered as a threat to pregnant women and their children’s health^1^. Exposure to secondhand smoke (SHS) is associated with cardiovascular or pulmonary diseases, malignancy, and numerous other health problems^2^. It has been established that passive smoking during pregnancy may lead to health risks for both the mother and child^3^, including pregnancy complications such as pre-eclampsia, and poor fetal outcomes^4,5^ such as low birthweight, premature birth etc. The prevalence of childhood asthma^6^, fetal dysplasia and nervous system diseases^7^ is also noted to be higher in children whose mothers were exposed to SHS during pregnancy.

Cerebral palsy (CP) is a group of permanent disorders that cause activity limitation including movement and posture development. It can be attributed to non-progressive disturbances that occurred in the developing fetal brain. Motor disorders of cerebral palsy are often accompanied with perception, sensational disturbances, communication, cognition and behavior, by secondary musculoskeletal problems or by epilepsy^8^. As the most common pediatric motor disability, it is not only a significant lifetime disability for children but also linked to a shortened life expectancy^9^.

As CP is a syndrome that partly is due to a lesion that may have occurred in the developing brain during pregnancy – a period of important neurodevelopment – SHS exposure may be a risk factor for early neurological dysplasia. In our study, the association between SHS exposure during pregnancy and children’s CP was assessed.

## METHODS

### Participants

This observational study was carried out in Public Health Communities in Shandong Province, China. We collected information on children with CP who accepted treatment in hospitals or were newly diagnosed during the screening. Healthy children were selected by public health communities’ physical examinations from 32 communities. In total, 6576 mother–child pairs who received treatment at our hospitals and attended health checkups at public health community centers were selected. As we were interested in the role of SHS exposure during pregnancy as a risk factor for unexplained CP, we excluded children who were diagnosed with another central nervous system anomaly or infection, a genetic or chromosomal abnormality, or traumatic brain injury, which may substantially have increased the risk of CP. Singleton births were also limited in our analysis. Subsequently, 5655 mother–child pairs completed our questionnaires. There were 337 participants with missing information of SHS exposure and they were excluded from the current analysis, while 5318 participants were included for final analysis, of which 5067 were non-smokers and were included. The study protocol was approved by the ethics committee of Liaocheng People’s Hospital, Shandong Provincial Hospital Affiliated to Shandong University, Qingdao Hiser Medical Group, and by the Liaocheng Public Health Communities. All participants gave written informed consent.

### Measurements of the exposure variable

A standardized questionnaire was adapted from the Global Tobacco Adult Survey. In order to describe smoking status during pregnancy, two options were provided to mothers: ‘never smoking’ and ‘ever smoking’. ‘Never smokers’ were defined as mothers who chose ‘never smoking’ and reporting no smoking in the past 30 days before pregnancy. SHS exposure was assessed by the question: ‘How many days have people smoked near you in one week during your pregnancy?’. The response options for exposure level were: 0, 1–4 , and 5–7 days/week. Definition of selfreported SHS exposure was non-smokers inhaled the smoke exhaled from smokers on at least 1 day/week. Women who replied ‘none’ or ‘0 day/week’ to this question were considered as ‘unexposed’ and others who chose ‘1–4 days/week’ or ‘5–7 days/week’ were considered as ‘exposed’. We also recorded other characteristics including childbearing age, household registration (location), and mode of delivery.

### Statistical analysis

Baseline characteristics were tabulated by children’s health status (CP or healthy). Continuous variables were summarized as mean ± standard deviation (SD). Differences in means for continuous variables among participants for different groups were calculated by Student’s t-tests. Categorical variables were presented as proportion (%) and compared using chi-squared tests. Logistic regression analyses were performed to estimate odds ratios (ORs) and 95% confidence intervals (95% CIs) of the association between different levels of exposure to SHS and risk of CP. All the statistical tests were two-tailed, and the cutoff significant level was defined as p<0.05. We performed all statistical analyses in R (version 3.6.1 https://cran.r-project.org).

## RESULTS

A total of 5318 mother–child pairs were collected in our study after excluding those with missing information. In order to restrict our study to the impact of SHS, we excluded the 251 actively smoking mothers from the final analysis, hence 5067 mother– child pairs were analyzed. Table 1 depicts the basic characteristics according to mother–child pairs participating in our study. Those who were exposed to SHS during pregnancy had a vaginal delivery and a lighter birthweight child were more frequent among pairs with CP at the bivariate level.

Table 2 shows the prevalence of SHS and its distribution characteristics according to our survey. The prevalence of SHS exposure of non-smokers during pregnancy was 72.3%. Mothers who did not work and lived in rural areas had a higher prevalence of SHS exposure, younger mothers were more frequently exposed to SHS, and the child’s weight at birth was lower than for those who were not exposed (p<0.05).

**Figure 1:**
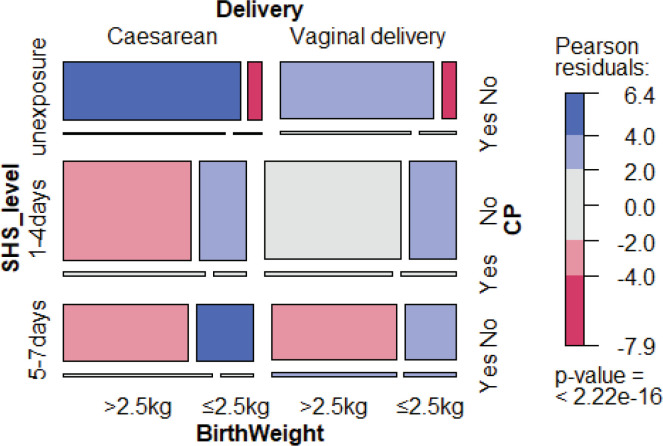
Mosaic of secondhand smoke exposure level, mode of delivery and birthweight

Figure 1 shows the distributions of the main characteristics of our study. Of the CP children’s mothers, 80.3% were exposed to SHS during their pregnancy, including 44.8% for 1–4 days/week and 35.5% for 5–7 days/week. For the 4828 children without CP, 71.9% of the mothers reported SHS exposure during pregnancy, including 44.4% for 1–4 days/week and 27.5% for 5–7 days/week (Table 3). Women who were exposed to SHS during pregnancy had higher odds of having a baby with CP (OR=1.60; 95% CI: 1.5–2.21) than those who were never exposed to SHS, in the crude logistic regression model. The association for SHS exposure during pregnancy and CP children remained significant when adjusting for mode of delivery and infant’s birthweight, due to their significant association with CP, with an OR of 1.44 (95% CI: 1.02–2.04) and AOR of 1.46 (95% CI: 1.13–1.91) for 1–4 days/week exposure; and an OR of 1.85 (95% CI: 1.28–2.66) and AOR of 1.63 (95% CI: 1.22–2.01) for 5–7 days/week exposure. In both crude and adjusted models, pregnant women that were more exposed to SHS had higher odds of having CP babies compared with mothers unexposed to SHS (Table 4).

**Table 1:** Characteristics of the study participants according to celebral palsy (CP) status, Shandong, 2017–2019 (N=5067)

*Characteristics*	*CPn (%)*	*No CP n (%)*	*p*
**Age of mother during pregnancy** (years)			
mean ± SD	30.33 ± 6.52	30.35 ± 5.88	0.96
**Occupation**			
House wife	123 (51.5)	2429 (50.3)	0.778
Employed	116 (48.5)	2399 (49.7)	
**Household registration**			
Urban	116 (48.5)	2467 (51.1)	0.479
Rural	123 (51.5)	2361 (48.9)	
**SHS exposure**			
No	47 (19.7)	1356 (28.1)	0.006
Yes	192 (80.3)	3472 (71.9)	
**Delivery mode**			
Vaginal delivery	137 (57.3)	2367 (49.0)	0.015
Caesarean section	102 (42.7)	2461 (51.0)	
**Infant’s sex**			
Girl	123 (51.5)	2301 (47.7)	0.279
Boy	116 (48.5)	2527 (52.3)	
**Infant’s birthweight** (kg)			
mean ± SD	3.05 ± 0.56	3.19 ± 0.68	0.0004

**Table 2:** Characteristics of the study participants according to SHS exposure, Shandong, 2017–2019 (N=5067)

*Characteristics*	*Exposed n (%)*	*Unexposed n (%)*	*p*
**Age of mother during pregnancy** (years)			
mean ± SD	29.74 ± 5.90	31.94 ± 5.64	<0.001
**Occupation**			
House wife	1997 (54.5)	555 (39.6)	<0.001
Employed	1667 (45.5)	848 (60.4)	
**Household registration**			
Urban	1672 (45.6)	911 (64.9)	<0.001
Rural	1992 (54.4)	492 (35.1)	
**Child status**			
No CP	3472 (94.8)	1356 (96.7)	0.006
CP	192 (5.2)	47 (3.3)	
**Delivery mode**			
Vaginal delivery	1846 (50.4)	658 (46.9)	0.029
Caesarean section	1818 (49.6)	745 (53.1)	
**Infant’s sex**			
Girl	1752 (47.8)	672 (47.9)	0.984
Boy	1912 (52.2)	731 (52.1)	
**Infant’s birthweight** (kg)			
mean ± SD	3.12 ± 0.71	3.34 ± 0.54	<0.001

**Table 3:** Characteristics of the study participants according to different SHS exposure levels (days/week), Shandong, 2017–2019 (N=5067)

*Characteristics*	*Unexposed (n=1403) n (%)*	*1–4 days (n=2250) n (%)*	*5–7 days (n=1414) n (%)*	*p*
**Age of mother during pregnancy** (years)				
mean ± SD	31.94 ± 5.64	29.70 ± 5.62	29.79 ± 6.31	<0.001
**Occupation**				
House wife	555 (39.6)	1097 (48.8)	900 (63.6)	<0.001
Employed	848 (60.4)	1153 (51.2)	514 (36.4)	
**Household registration**				
Urban	911 (64.9)	1155 (51.3)	517 (37.6)	<0.001
Rural	492 (35.1)	1095 (48.7)	897 (63.4)	
**Child status**				
No CP	1356 (96.7)	2143 (95.2)	1329 (94.0)	0.004
CP	47 (3.3)	107 (4.8)	85 (6.0)	
**Delivery mode**				
Vaginal delivery	658 (46.9)	1148 (51.0)	698 (49.4)	0.053
Caesarean section	745 (53.1)	1102 (49.0)	716 (50.6)	
**Infant’s sex**				
Girl (%)	672 (47.9)	1057 (47.0)	695 (49.2)	0.439
Boy (%)	731 (52.1)	1193 (53.0)	719 (50.8)	
**Infant’s birthweight** (kg)				
mean ± SD	3.34 ± 0.54	3.14 ± 0.71	3.09 ± 0.72	<0.001

**Table 4:** Prevalence of SHS exposure in non-smoker pregnant mother and child, and OR (95% CI) of CP according to mother’s exposure to SHS during pregnancy, Shandong, 2017–2019 (N=5067)

*SHS exposure (days/week)*	*Total*	*Child health status*		*OR (95% CI)*	*AOR (95% CI)*
	*n*	*CP n (%)*	*No CP n (%)*		
0 (Ref.)	1403	47 (19.7)	1356 (28.1)	1	1
1–4	2250	107 (44.8)	2143 (44.4)	1.44 (1.02–2.04)	1.46 (1.13–1.91)
5–7	1414	85 (35.5)	1329 (27.5)	1.85 (1.28–2.66)	1.63 (1.22–2.01)
All	5067	239	4828	1.60 (1.15–2.21)	1.44 (1.07–1.89)

OR: odds ratio. CI: confidence interval. SHS: secondhand smoke. AOR: adjusted odds ratio, different exposure sources were adjusted mutually and for birthweight and delivery. CP: celebral palsy.

## DISCUSSION

Our study provides an insight into the association between maternal SHS exposure during pregnancy and children’s CP. We found that SHS exposure during pregnancy was associated with an increased risk of CP. When we adjusted for confounding factors in the analysis, the exposure-outcome relationship remained statistically significant.

Tobacco use induces human disease and causes an economic burden worldwide^10-12^. SHS consists of 85% sidestream and 15% exhaled mainstream smoke^13^, hundreds of metal ingredients, and enduring harmful chemical gases suspended in the available breathing environment. A report from the US Centers for Disease Control and Prevention showed that nearly 50% of US non-smokers are exposed to SHS^14^, and there is no risk-free level of SHS exposure^10,15^. In developing countries, around 70% of men and 3% of women^16^ smoke. In China, the prevalence of active and passive smoking^17^ are both at high levels partly due to the failure of tobacco control enforcement measures in public areas. In our study, 4.7% of women smoked, and the SHS exposure prevalence of nonsmoker pregnant women was 72.3%, which is a similar prevalence to that in Indonesia (76%)^18^. We also found that women who live in rural areas and did not work had a higher prevalence of SHS exposure, which we hypothesize is closely related to their economic and educational level.

Passive inhalation of tobacco smoke and its byproducts could impact brain development and also increase the risk of dementia syndromes and mortality after stroke^19,20^. More than 41 million deaths among non-smoker adults and 400 deaths in infants are caused by exposure to SHS each year^21^. Consequences are very serious when a mother stays in the environment where tobacco smoke is present during pregnancy. Household wife’s exposure to SHS is higher among younger individuals^20^, and our study also showed that younger people were more frequently exposed to SHS. Since most pregnant women, who constitute a vulnerable population, are aged 20–40 years and typically spend more time indoors, it is not easy for them to avoid SHS exposure. Additionally, increased abdominal size and respiration, changes of immunologic systems and metabolic capacity^22^ of pregnant women may make them more sensitive than others to pollutants. Recently, studies found that SHS may represent a developmental neurotoxic during gestation^23,24^, and the brain’s executive system can be ultimately influenced by neurotoxicity during critical periods in brain development^19^. A study reported that secondhand smoke can affect the central neuroexcitatory^25^. It is well established that mothers’ smoking during pregnancy is associated with higher neurological risks in development and growth^26^. There are many other noxious compounds in tobacco smoke that can cross the placental barrier and influence prenatal development^27^. A metaanalysis showed that women exposed to SHS during pregnancy is associated with higher risk of infants’ neural tube defects^28^.

A systematic review^29^ reported several risk factors such as placental problems, birth defects, low birthweight, emergency caesarean section, respiratory distress syndrome, birth asphyxia, and neonatal infections etc., as significantly associated with CP. In our study, we found that SHS exposure during pregnancy increased the risk of children’s CP compared with those without these exposures and the risk increased with the exposure time. Previous research has indicated an association between SHS and low birthweight^30^. Prenatal exposure to SHS has higher risk of stillbirth, congenital malformation^31^, and lower birthweight^32-35^. SHS exposure during pregnancy is associated with lower birthweight in our study, birthweight became lower when mothers were exposed to increasing doses of SHS, from unexposed to exposure 5–7 days/week; this can be another pathway from SHS to CP. We also found dose–response relations between increased risk of CP and duration of SHS exposure. We were unable to compare our results with the other studies because no previous studies on CP children evaluated the SHS exposure duration. Our study builds on the literature on SHS exposure during pregnancy to assess the setting-specific association between prenatal SHS exposure and children’s CP.

Comprehensive tobacco control measures have been enacted in China these years, and the smoke-free areas were greatly extended to most indoor public places. However, a substantial proportion of pregnant women are still exposed to tobacco smoke where there is no smoking. Although multiple factors play a role in CP, there remain some risk factors that can be circumvented through public health strategies. The prevention of SHS exposure represents a modifiable risk factor for preventing CP.

### Strengths and limitations

The strengths of our study are that we have a large sample size from three cities in Shandong Province, China and we explored the dose–response relationship between SHS exposure level and CP. Furthermore, self-assessment surveys by mothers were performed in hospital and clinical service departments in the community, so the data are more reliable when collected by professional neurosurgeons and pediatricians. However, potential limitations also need to be considered. First, this study was an observational study and is more likely to be affected by confounding factors; therefore, although we demonstrated that exposure to SHS during pregnancy possibly causes children’s CP, we did not clinically measure exposure to SHS in this study. Second, our data may not be representative of all pregnant women in China. Third, although the strength of this study is the dose–response of SHS exposure, it is limited by self-report and may need biochemical measurements to ascertain further mechanisms.

## CONCLUSIONS

Our study suggests an association between prenatal SHS exposure and CP. The finding enhances our understanding of other risk factors for CP and emphasizes the necessity to protect pregnant women and avoid CP. Actions should be implemented to reduce the harmful effects of SHS exposure in mothers and their babies during pregnancy.
